# Preoxygenation With and Without Positive End-Expiratory Pressure in Lung-Healthy Volunteers: A Randomized Clinical Trial

**DOI:** 10.1001/jamanetworkopen.2025.11569

**Published:** 2025-05-20

**Authors:** Giulia Roveri, Anna Camporesi, Alex Hofer, Simon Kahlen, Franziska Breidt, Simon Rauch

**Affiliations:** 1 Institute of Mountain Emergency Medicine, Eurac Research, Bolzano, Italy; 2 Department of Anesthesia and Intensive Care Medicine, Merano Hospital, Merano, Italy; 3 Department of Pediatric Anesthesia and Intensive Care Unit, Buzzi Children’s Hospital, Milan, Italy; 4 Aiut Alpin Dolomites HEMS (Helicopter Emergency Medical Services), Pontives, Italy; 5 Medical Faculty of University Ulm, Ulm, Germany; 6 Department of Anaesthesiology, Heidelberg University Hospital, Heidelberg, Germany

## Abstract

**Importance:**

Optimal preoxygenation is critical in emergency medicine to prevent desaturation during airway management, especially in high-risk populations. Identifying the most effective preoxygenation device across diverse patient groups remains a clinical priority.

**Objective:**

To compare the efficacy of 3 preoxygenation devices—nonrebreather mask (NRM), bag-valve mask (BVM), and BVM with positive end-expiratory pressure (BVM plus PEEP)—in lung-healthy volunteers.

**Design, Setting, and Participants:**

This crossover randomized clinical trial was conducted from May 26 to 31, 2024, at Eurac Research, Bolzano, Italy. Volunteer participants included lung-healthy adults with normal weight (NM), adults with overweight or obesity (OW-OB), and children aged 6 to 12 years.

**Exposures:**

Preoxygenation using each device (NRM, BVM, BVM plus PEEP) with 15 L/min of oxygen for a duration of 3 minutes in a randomized order.

**Main Outcomes and Measures:**

The primary outcome was expiratory oxygen concentration (Feo_2_) at the end of preoxygenation. Secondary outcomes included ventilation in dependent lung regions assessed using electrical impedance tomography, noninvasive continuous monitoring of oxygenation status (oxygenation reserve index [ORI]) at the end of preoxygenation, and the time taken for ORI to return to baseline values.

**Results:**

The study included 53 participants, 39 male (74%) and 14 female (26%), of whom 16 were adults with NW (mean [SD] age, 36 [11] years), 18 were adults with OW-OB (mean [SD] age, 45 [11] years), and 19 were children (mean [SD] age, 8 [3] years). Mean (SD) Feo_2_ at the end of preoxygenation was higher with BVM and BVM plus PEEP compared with NRM in adults with NW (72.1% [5.9%] and 75.6% [4.3%], respectively, vs 52.5% [6.1%]; *P* < .001), adults with OW-OB (65.8% [10.4%] and 73.0% [6.4%], respectively, vs 51.9% [6.1%]; *P* < .001), and children (64.6% [13.4%] and 67.5% [10.2%], respectively, vs 38.5% [7.5%]; *P* < .001). Ventilation in dependent lung regions was higher with BVM plus PEEP than NRM in adults with NW (BVM plus PEEP, 51.9 [9.3] vs NRM, 47.0 [5.7]; *P* = .03) and children (BVM plus PEEP, 53.0 [7.3] vs NRM, 47.7 [7.0]; *P* = .002). ORI at the end of preoxygenation was higher with BVM plus PEEP than with NRM in adults with OW-OB (BVM plus PEEP, 0.79 [0.13] vs NRM, 0.73 [0.13]; *P* < .001). Additionally, the mean (SE) time for ORI to return to baseline was longer with BVM plus PEEP compared with NRM in both adults with OW-OB (BVM plus PEEP, 196 [74] seconds vs NRM, 158 [53] seconds; *P* = .01) and children (BVM plus PEEP, 115 [59] seconds vs NRM, 62 [36] seconds; *P* < .001).

**Conclusions and Relevance:**

In this crossover randomized clinical trial, preoxygenation with PEEP was more effective than preoxygenation without PEEP, resulting in higher Feo_2_ values and improved ventilation in dependent lung regions. These findings suggest that BVM plus PEEP should be prioritized for preoxygenation in emergency settings.

**Trial Registration:**

ClinicalTrials.gov Identifier: NCT06370689

## Key Points

**Question:**

Is there a difference in the efficacy of 3 preoxygenation devices for use in emergency medicine—nonrebreather mask (NRM), bag-valve mask (BVM), and BVM with positive end-expiratory pressure (BVM plus PEEP)—in lung-healthy volunteers?

**Findings:**

In this crossover randomized clinical trial of 53 volunteers, including adults with normal weight, adults with overweight or obesity, and children aged 6 to 12 years, BVM and BVM plus PEEP achieved higher expiratory oxygen concentration compared with NRM. BVM plus PEEP improved ventilation in dependent lung regions.

**Meaning:**

These findings suggest that adding PEEP to BVM may improve preoxygenation of patients undergoing emergency intubation.

## Teaser

This crossover randomized clinical trial evaluates the efficacy of nonrebreather mask, bag-valve mask, and bag-valve mask with positive end-expiratory pressure oxygenation devices among lung-healthy volunteers consisting of adults with normal weight, adults with overweight or obesity, and children aged 6 to 12 years.

## Introduction

Hypoxemia is a frequent and potentially life-threatening complication during intubation of critically ill patients, potentially leading to severe outcomes such as cardiovascular collapse, anoxic brain injury, or death.^1^ Effective preoxygenation is crucial to extend the safe apneic period and mitigate the risk of hypoxemia, especially in cases of unanticipated difficult airway management. The primary goal of preoxygenation is to maintain arterial oxygen saturation (Sao_2_) during apnea despite ongoing oxygen consumption. This is achieved by denitrogenating the alveoli, allowing the lung’s functional residual capacity (FRC) to act as an oxygen reservoir.^2^

Various preoxygenation strategies exist, but their application is highly variable,^1,3^ with current guidelines often lacking specific recommendations^4^ or defaulting to facemask use.^5,6^ Nonrebreather facemasks (NRMs) and bag-valve masks (BVMs) without positive end-expiratory pressure (PEEP) are the most commonly used techniques in both hospital and prehospital settings.^1,3^ However, evidence from the operating room, intensive care unit, and emergency department consistently demonstrates that preoxygenation with PEEP improves oxygenation and extends the safe apnea period during intubation.^7,8,9^ A recent study in critically ill patients in the intensive care unit and emergency department^10^ further highlighted that noninvasive ventilation (NIV) with PEEP reduces the incidence of hypoxemia compared to NRMs. However, NIV is often unavailable for preoxygenation in the prehospital setting. In this context, intubation is especially challenging and peri-intubation hypoxemia occurs in as many as 20% of cases.^11^ Evidence on the most effective preoxygenation method in the prehospital setting is limited,^12,13^ especially for high-risk groups such as children and adults with obesity (OB), who are predisposed to rapid oxygen desaturation during apnea due to elevated oxygen consumption and significantly reduced FRC.^2,11,14,15^

This study aimed to evaluate the efficacy of 3 preoxygenation devices—NRM, BVM, and BVM with a PEEP valve (BVM plus PEEP)—across 3 participant groups: adults with normal weight (NW), adults with overweight or OB (OW-OB), and children aged 6 to 12 years. We hypothesized that adding PEEP during preoxygenation would improve efficacy, particularly in children and individuals with OB, by improving ventilation in the dependent lung regions.

## Methods

### Study Design and Setting

This interventional, crossover randomized clinical trial was conducted in an ambulatory care room at Eurac Research, Bolzano, Italy, from May 26 to 31, 2024. The study protocol was approved by the Ethics Institutional Review Board for Clinical Studies of Bolzano and prospectively registered before recruitment of the first participant (Supplement 1). All volunteers gave written informed consent to participate in the study. For participants aged 6 to 12 years, written informed consent was obtained from parents or legal guardians before study participation. The children were also provided with an aged-tailored brochure for the intervention. The study was conducted in accordance with Good Clinical Practice and followed the Declaration of Helsinki Guidelines.^16^ The study is reported according to the Consolidated Standards of Reporting Trials Extension (CONSORT Extension) reporting guideline (eFigure in Supplement 2).^17,18^

### Participants

For adults with NW, the inclusion criteria consisted of a body mass index (BMI; calculated as the weight in kilograms divided by the height in square meters) ranging from 18 to 24 with an American Society of Anesthesiologists physical status score (ASA) of 1 or 2. For adults with OW-OB,^19^ these consisted of a BMI of 25 or greater and an ASA of 3 or less. For children aged 6 to 12 years, inclusion criterion was an ASA of 1.^19,20^ Exclusion criteria were history of smoking, acute respiratory illness on the study day, and pregnancy.

### Exposure and Measurements

Each participant underwent 3 preoxygenation sessions in a crossover design, using 1 of 3 devices per session: NRM, BVM, and BVM plus PEEP. A computer-generated randomization list with permutation blocks ensured a balanced allocation of participants to each permutation. Participants were blinded to the session sequence, and a 30-minute washout period between sessions was implemented to mitigate carryover effects.

Each preoxygenation session consisted of 3 minutes of spontaneous tidal volume breathing using the assigned device. For the NRM, investigators optimized the mask position by adjusting the elasticated headband. For the BVM and BVM plus PEEP groups, a BVM with a built-in nonrebreathing valve (Ambu Mark IV; Ambu A/S) was used to ensure fresh oxygen flow to the patient while preventing rebreathing of exhaled gases. A single investigator (G.R.) ensured a secure and tight mask seal throughout the session by firmly holding the mask in place without providing assisted ventilation. For the BVM plus PEEP technique, PEEP was set at 8 cm H_2_O for adults and 5 cm H_2_O for children, with the characteristic hissing sound from the valve confirming a proper mask seal. Oxygen was supplied via standard wall flowmeters at a flow rate of 15 L/min for all devices.

Before the preoxygenation phase, participants completed a 3-minute baseline phase in the sitting position, followed by a 3-minute baseline phase in the supine position. Measured parameters included peripheral oxygen saturation (Spo_2_), heart rate, respiratory rate, expired oxygen concentration (Feo_2_), electrical impedance tomography (EIT), and noninvasive continuous monitoring of oxygenation status (Oxygen Reserve Index; Masimo Corporation), with values reported as oxygenation reserve index (ORI).

The mean single-breath FeO_2_ was measured using the first breath after preoxygenation, with participants exhaling into a balloon connected to a calibrated gas analyzer (Dräger X-am 8000; Dräger Safety AG & Co). EIT was used for real-time regional lung ventilation assessment.^21^ A dedicated belt was placed transversely in the fourth and fifth intercostal space^22^ and connected to a commercial EIT monitor (ENLIGHT 2100; Timpel Medical). Small alternating electrical currents were applied around the participant’s thorax to generate EIT data, which were stored for offline analysis. The imaging field was divided into 2 equal-sized contiguous regions of interest: the nondependent (upper half) and dependent (lower half) regions.^21,22^

The ORI, a dimensionless index ranging from 0 to 1 that reflects oxygenation in the moderate hyperoxic range, was measured using a fingertip sensor (RD Rainbow Lite SET-1; Masimo Corporation) and oximeter (Radical-7 Pulse CO-Oximeter; Masimo Corporation). When pure oxygen is administered, Sao_2_ reaches 100% at a partial pressure of oxygen (Pao_2_) of 100 mm Hg. Beyond this point, as Pao_2_ continues to rise, Sao_2_ and SpO_2_ remain at 100%, and the ORI increases nonlinearly from 0.00 (Pao_2_ approximately 100 mm Hg) to 1.00 (Pao_2_ approximately 200 mm Hg).^23,24^

Measurements were taken at the end of the 3-minute baseline phase in a sitting position, at the end of the 3-minute baseline phase in the supine position, and at the end of the preoxygenation phase. Regional distribution of ventilation was additionally recorded at 1.5 minutes into the preoxygenation phase. For ORI, the time taken for ORI to return to baseline values (ORI at the end of the baseline phase in the supine position) following preoxygenation was also measured.

### Outcomes

The primary outcome was the difference in Feo_2_ at the end of the preoxygenation phase among the 3 devices. Secondary outcomes included differences in regional ventilation within the dependent lung regions at the end of the preoxygenation phase, changes in regional ventilation in the dependent lung regions from the end of baseline in a sitting position to the end of baseline in a supine position and from the end of baseline in a supine position to the end of the preoxygenation phase, differences in ORI at the end of the preoxygenation phase, and differences in the time taken for ORI to return to baseline values (ORI at the end of baseline in a supine position) following preoxygenation.

### Sample Size Calculation

The sample size was calculated based on an estimated effect size of 10 and an SD of 8, derived from a previous study.^25^ Using a power of 0.8 and a significance level of 0.05, and accounting for the repeated measures design with an intraclass correlation (ρ value) of 0.3, the required sample size was determined to be 15 participants per group.

### Statistical Analysis 

All statistical analyses were performed using Stata, version 18 BE (StataCorp LLC). A 2-sided *P* < .05 was considered statistically significant. Baseline characteristics were summarized using descriptive statistics. Changes in primary and secondary outcomes over time were analyzed across groups (adults with NW, adults with OW-OB, and children) using linear mixed-effects models, with fixed effects for time points, device (NRM, BVM, and BVM plus PEEP), and device permutation to account for potential carryover effects. A random effect on the participant addressed data dependency. Pairwise comparisons of marginal means were used to evaluate differences between devices, with 95% CIs and *P* values computed for each contrast. No data were missing.

## Results

The study included 53 participants: 39 male (72%) and 14 female (28%). Among them, 16 were participants with NW and a mean (SD) age of 36 (11) years, 18 were adults with OW-OB and a mean (SD) age of 45 (11) years, and 19 were children with a mean (SD) age of 8 (3) years. All 53 participants completed the study with no missing data. Demographic data and baseline characteristics are summarized in Table 1.

**Table 1.** Demographic Data and Baseline Characteristics of the Participants

Characteristic	Participant group, mean (SD)		
	Adults with normal weight (n = 16)	Adults with overweight or obesity (n = 18)	Children aged 6-12 y (n = 19)
Age, y	36 (11)	45 (11)	8 (3)
Gender, No. (%)			
Female	9 (56)	1 (6)	4 (21)
Male	7 (44)	17 (94)	15 (79)
Weight, kg	64.5 (11.3)	103.7 (20.4)	30.5 (14.7)
Height, cm	172.6 (9.0)	179.5 (6.9)	134.4 (16.2)
BMI	21.5 (1.9)	32.2 (6.3)	16.8 (3.4)

Abbreviation: BMI, body mass index (calculated as the weight in kilograms divided by the height in square meters).

### Feo_2_ at the End of the Preoxygenation Phase

**Figure 1.**
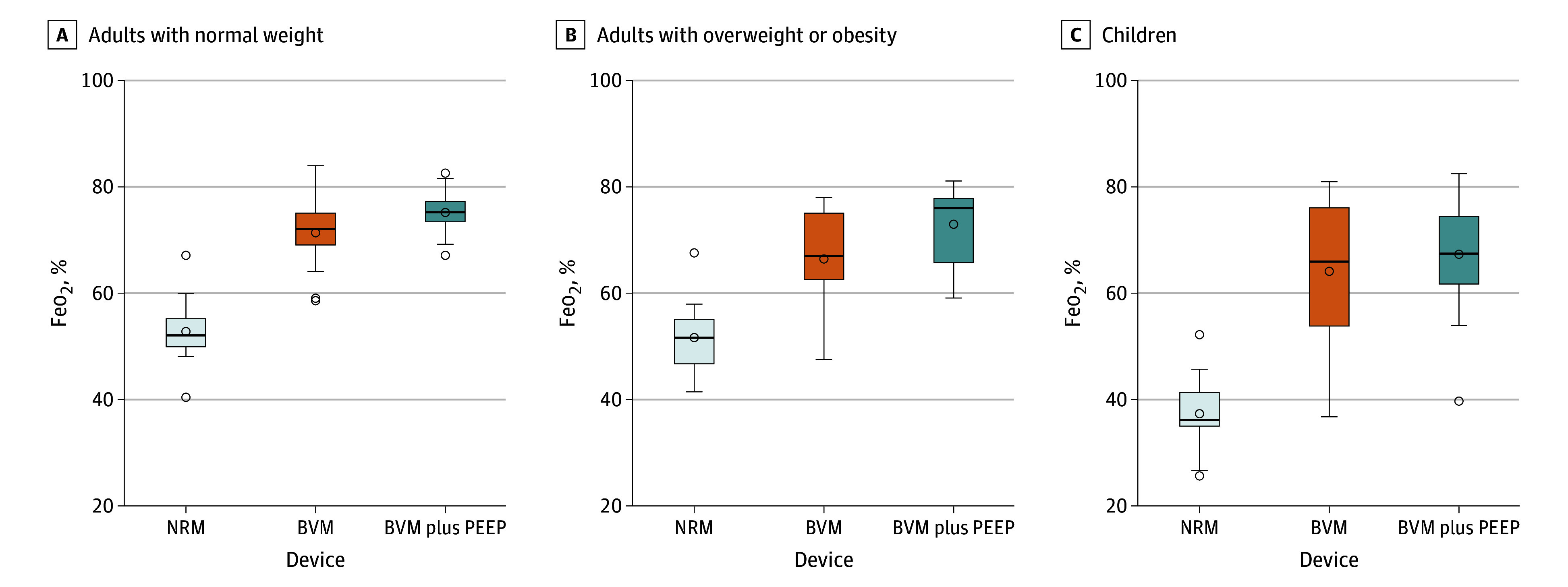
Differences in Expired Oxygen Concentration (Feo_2_) at End of Preoxygenation Results are shown across nonrebreather facemask (NRM), bag-valve mask (BVM), and BVM plus positive end-expiratory pressure (BVM plus PEEP) devices in adults with normal weight, adults with overweight or obesity, and children aged 6 to 12 years. Boxes indicate 95% CIs; dots, means; and horizontal bars, median.

Mean Feo_2_ values at the end of the preoxygenation phase for all groups and devices are presented in Figure 1 and Table 2, with pairwise comparisons detailed in eTable 1 in Supplement 2. Mean (SD) Feo_2_ at the end of preoxygenation was higher with BVM and BVM plus PEEP compared with NRM in adults with NW (72.1% [5.9%] and 75.6% [4.3%], respectively, vs 52.5% [6.1%]; *P* < .001), adults with OW-OB (65.8% [10.4%] and 73.0% [6.4%], respectively, vs 51.9% [6.1%]; *P* < .001), and children (64.6% [13.4%] and 67.5% [10.2%], respectively, vs 38.5% [7.5%]; *P* < .001). In adults (NW and OW-OB groups), the difference in Feo_2_ with BVM plus PEEP was significantly higher than with BVM without PEEP (BVM plus PEEP vs BVM without PEEP, 75.6 [4.3] vs 72.1 [5.9]; *P* = .03 in NW and 73.0 [6.4] vs 65.8 [10.4]; *P* = .005 in OW-OB, respectively).

**Table 2.** Measurements During Preoxygenation Sessions Using 3 Study Devices

Measurement by device	Participant group, mean (SD)		
	Adults with normal weight	Adults with overweight or obesity	Children
Feo_2_ at T-Preox, %			
NRM	52.5 (6.1)	51.9 (6.1)	38.5 (7.5)
BMV	72.1 (5.9)	65.8 (10.4)	64.6 (13.4)
BMV plus PEEP	75.6 (4.3)	73.0 (6.4)	67.5 (10.2)
Ventilation in the dependent lung regions at T-BL-Sit, %			
NRM	52.8 (6.0)	57.1 (11.1)	56.6 (9.5)
BMV			
BMV plus PEEP			
Ventilation in the dependent lung regions at T-BL-Sup, %			
NRM	47.8 (5.5)	43.9 (8.2)	48.5 (8.2)
BMV	48.3 (5.9)	46.3 (9.7)	47.7 (7.8)
BMV plus PEEP	45.7 (4.4)	46.5 (9.7)	48.0 (4.2)
Ventilation in the dependent lung regions at T-Preox, %			
NRM	47.0 (5.7)	43.4 (8.4)	47.7 (7.0)
BMV	49.0 (7.2)	44.8 (6.9)	50.9 (7.6)
BMV plus PEEP	51.9 (9.3)	48.7 (6.9)	53.0 (7.3)
ORI at T-Preox			
NRM	0.8 (0.1)	0.7 (0.1)	0.5 (0.1)
BMV	0.7 (0.1)	0.7 (0.1)	0.6 (0.1)
BMV plus PEEP	0.8 (0.1)	0.7 (0.1)	0.6 (0.1)
Time to return ORI back to baseline, s			
NRM	208 (94)	158 (53)	62 (36)
BMV	211 (102)	186 (70)	83 (51)
BMV plus PEEP	227 (81)	196 (74)	115 (59)

Abbreviations: BMV, bag-valve mask; BVM plus PEEP, BVM with positive end-expiratory pressure; Feo_2_, expiratory oxygen concentration; NRM, nonrebreather facemask; T-BL-Sit, end of baseline in a sitting position; T-BL-Sup, end of baseline in a supine position; T-Preox, end of preoxygenation

### Ventilation in Dependent Lung Regions

**Figure 2.**
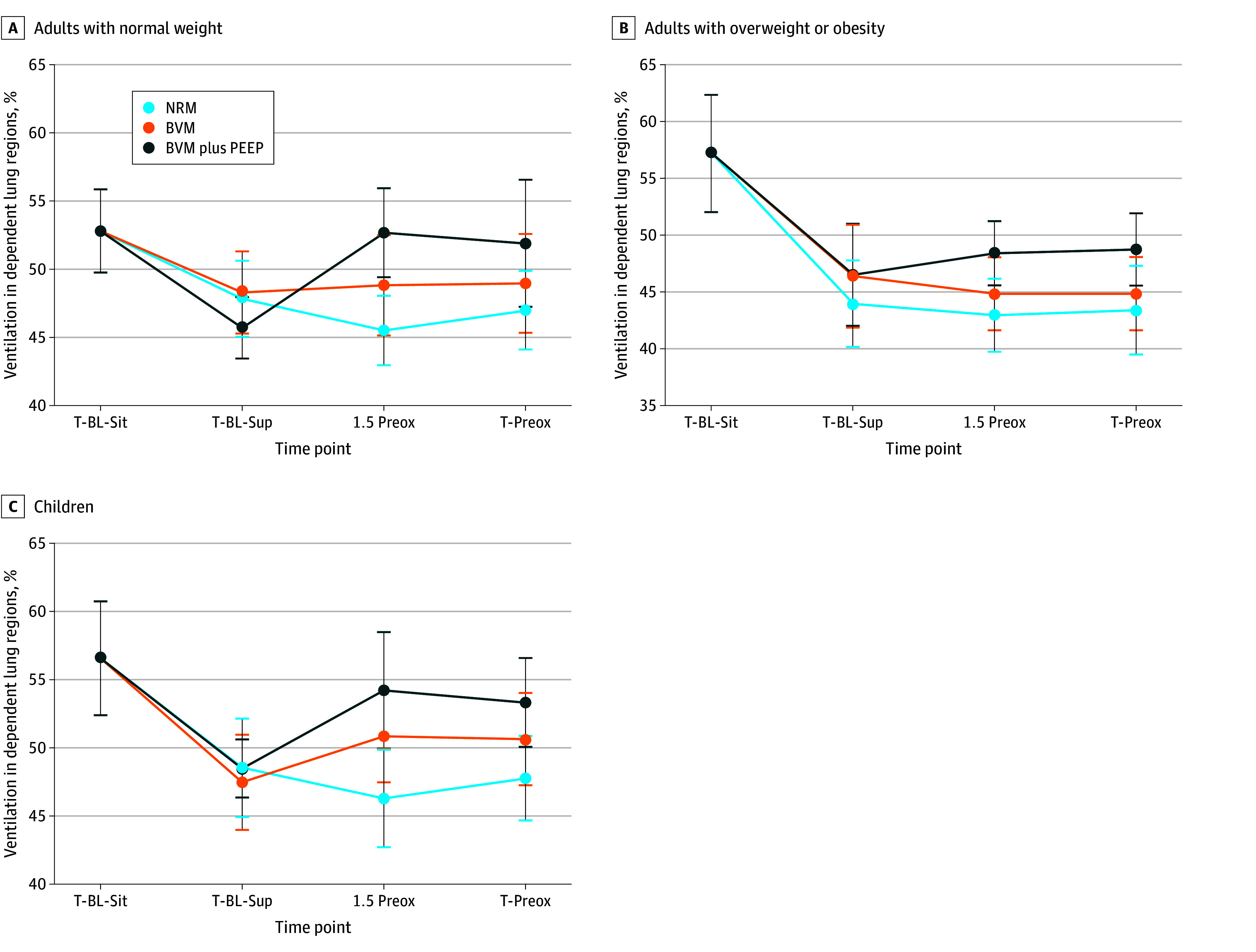
Ventilation in the Dependent Lung Regions Measured by Electrical Impedance Tomography at Different Time Points Data are expressed as mean (95% CI). BMV indicates bag-valve mask; BVM plus PEEP, BVM with positive end-expiratory pressure; NRM, nonrebreather facemask; T-BL-Sit, end of baseline in a sitting position; T-BL-Sup, end of baseline in a supine position; T-Preox, end of preoxygenation; and 1.5 Preox, 1.5 minutes into preoxygenation.

Figure 2 and Table 2 illustrate the percentage of ventilation in the dependent lung regions across all groups at different time points for each device. During the transition from sitting to supine positions, ventilation in dependent lung regions decreased in all groups: −5.0% (95% CI, −8.4% to −1.6%; *P* = .004) in the NW group, −12.9% (95% CI, −16.9% to −8.9%; *P* < .001) in the OW-OB group, and −8.1% (95% CI, −11.7% to −4.4%; *P* < .001) in the pediatric group. During preoxygenation (from end of baseline in the supine position to end of preoxygenation), ventilation in the dependent lung regions remained unchanged with NRM (Figure 2 and Table 2). In contrast, ventilation significantly increased with BVM plus PEEP in the NW group (mean [SD] values, 45.7 [4.4] in BL sup vs 51.9 [9.3] at end of preoxygenation; *P* = .004) and in the pediatric group (mean [SD] values, 48.0 [4.2] in BL sup vs 53.0 [7.3] at end of preoxygenation; *P* = .007), while the increase was not statistically significant in the OW-OB group (mean [SD] values, 46.5 [9.7] in BL sup vs 48.7 [6.9] at end of preoxygenation; *P* = .28). At the end of preoxygenation, differences between the mean (SE) ventilation values in the dependent lung regions were significantly higher with BVM plus PEEP compared with NRM in the NW group (BVM plus PEEP, 51.9 [9.3] vs NRM; 47.0 [5.7]; *P* = .03) and the pediatric group (BVM plus PEEP, 53.0 [7.3] vs NRM, 47.7 [7.0]; *P* = .002), and higher with BVM plus PEEP than BVM without PEEP in the OW-OB group (BVM plus PEEP, 48.7 [6.9] vs BVM without PEEP, 44.8 [6.9]; *P* < .001) and the NW group (BVM plus PEEP, 51.9 [9.3] vs BVM without PEEP, 49.0 [7.2]; *P* = .002) (eTable 2 in Supplement 2).

### ORI

Mean ORI values at the end of preoxygenation and the time for ORI to return to baseline values (end of baseline in the supine position) after preoxygenation across all groups and devices are presented in Table 2. Pairwise comparisons of ORI at the end of preoxygenation are shown in eTable 3 in Supplement 2, while comparisons of time needed for ORI to return to baseline are detailed in eTable 4 in Supplement 2. In the OW-OB group, the mean (SE) ORI at the end of preoxygenation was higher with BVM plus PEEP compared with both NRM (BVM plus PEEP, 0.79 [0.13] vs NRM, 0.73 [0.13]; *P* < .001) and BVM without PEEP (0.75 [0.13]; *P* = .04). No significant differences were observed in the NW and pediatric groups. The difference between the mean (SE) time for ORI to return to baseline values following preoxygenation was significantly longer with BVM plus PEEP than NRM in the OW-OB group (BVM plus PEEP, 196 [74] seconds vs NRM, 158 [53] seconds; *P* = .01) and longer with BVM plus PEEP compared to both NRM (BVM plus PEEP, 115 [59] seconds vs NRM, 62 [36] seconds; *P* < .001) and BVM without PEEP (83 [51] seconds; *P* = .008) in the pediatric group. No significant difference was observed in NW.

## Discussion

This crossover randomized clinical trial evaluated the effectiveness of 3 preoxygenation devices in emergency settings—NRM, BVM, and BVM plus PEEP—in adults with NW, adults with OW-OB, and children aged 6 to 12 years. A summary of findings follows.

### Methodological Considerations

While studies in the intensive care unit and emergency department have shown that NIV improves oxygenation and prolongs safe apnea during intubation,^7,8,9,10^ NIV is often unavailable in prehospital settings, where preoxygenation typically relies on facemasks or BVM without PEEP.^3^ Optimizing preoxygenation in these contexts is crucial, especially for patients with OW-OB and children, who experience desaturation more rapidly due to reduced FRC and increased oxygen consumption.^2,11^ Attaching a PEEP valve to the BVM offers a practical alternative when NIV is not accessible. However, direct comparisons among NRM, BVM, and BVM plus PEEP in these high-risk populations are scarce, and most studies rely solely on Feo_2_ as an outcome measure.^26^ This study addresses 4 key gaps: (1) comparing preoxygenation with BVM plus PEEP and the 2 most commonly used methods in emergency medicine—NRM and BVM without PEEP; (2) including high-risk populations such as children and adults with OW-OB, who are more prone to rapid oxygen desaturation; (3) assessing regional ventilation with EIT to investigate a key mechanism through which PEEP might enhance preoxygenation; and (4) incorporating the ORI as an outcome parameter, which may offer a more accurate reflection of arterial oxygen content compared with Feo_2_.

### Feo_2_

We used Feo_2_ as the primary outcome parameter, as it reflects the efficacy of denitrogenation and is the most common primary end point in preoxygenation studies.^2^ The alveoli achieve near-complete denitrogenation at 90% end-tidal oxygen (EtO_2_).^27^ However, we measured mixed single-breath Feo_2_, which is inherently lower than end-tidal values. This explains why the Feo_2_ levels in our study were lower than those reported in the anesthesia literature, where EtO_2_ is commonly used.^2^

The primary finding of our study was that both BVM and BVM plus PEEP were significantly more effective than NRM in achieving higher Feo_2_. These findings partially align with those of Groombridge et al,^25,26^ who reported that preoxygenation with BVM (without PEEP) resulted in higher Feo_2_ compared with NRM. Notably, our study expands on this by showing that in adults, BVM plus PEEP achieved even higher Feo_2_ levels than BVM alone, highlighting the additional oxygenation benefits of PEEP.

### Ventilation in Dependent Lung Regions

Studies conducted in the operating room have demonstrated that atelectasis in the dependent lung regions develops almost immediately after anesthesia induction, particularly in children and patients with OB, leading to decreased FRC and increased ventilation-perfusion mismatch.^28,29,30,31^ Preventing atelectasis in these regions during preoxygenation and anesthesia induction is crucial to reducing the risk of hypoxemia. Preoxygenation with a tight-fitting mask and 10-cm H_2_O PEEP temporarily increased FRC during anesthesia induction in patients with OB,^32^ whereas BMV without PEEP significantly reduced postintubation FRC.^33^ Based on the physiological principle that PEEP maintains or increases FRC by preventing atelectasis in dependent lung regions,^34^ we investigated the impact of different preoxygenation devices on lung aeration. Additionally, we examined the influence of body position on regional lung ventilation. To our knowledge, this is the first study to compare ventilation distribution during preoxygenation across different devices. We found that the transition from a sitting to a supine position significantly reduced ventilation in dependent lung regions, consistent with prior studies that have shown a position-dependent decrease in FRC when moving to the supine position.^35,36^ During preoxygenation with NRM, ventilation in these regions remained unchanged, highlighting the limitations of this common method. In contrast, preoxygenation with BVM plus PEEP increased ventilation in dependent regions across all groups, although the improvement was not statistically significant in adults with OW-OB, possibly due to insufficient PEEP levels. At the end of preoxygenation, ventilation in the dependent lung regions was higher with PEEP than without PEEP, highlighting its added benefits in preventing atelectasis in dependent lung regions.

### ORI

In addition to Feo_2_, we included the ORI as an outcome parameter. While Feo_2_ is widely used to evaluate preoxygenation effectiveness, it primarily reflects denitrogenation rather than arterial oxygen content. In patients with a high shunt fraction (eg, due to atelectasis), even maximal denitrogenation (high Feo_2_) may not sufficiently enhance arterial oxygen content when preoxygenation is performed without PEEP, potentially reducing the duration of safe apnea.^37^ ORI, which provides an index of arterial oxygen content, may offer a more comprehensive measure of preoxygenation efficacy in such cases.^23^ The ORI values obtained after preoxygenation in our study align with those reported in the literature for lung-healthy children and adults undergoing preoxygenation in the operating room.^38,39^ Our study found no significant differences in ORI among the 3 preoxygenation devices in the NW and pediatric groups. This is likely due to the inclusion of participants with healthy lungs and normal alveolar-arterial gradients, where shunting is minimal. In these individuals, even a fraction of inspired oxygen as low as 0.4—achievable with any of the 3 devices—can result in a Pao_2_ exceeding 200 mm Hg, producing uniformly high ORI values. However, we assume that in patients with significant shunting, the addition of PEEP could enhance arterial oxygen content and extend the duration of safe apnea.^7^ In the OW-OB group, ORI values after preoxygenation were higher with PEEP than without. This may be attributed to PEEP preventing atelectasis and thus reducing ventilation-perfusion mismatch in dependent lung regions. In the OW-OB and pediatric groups, preoxygenation with BVM plus PEEP prolonged the time for ORI to return to baseline by 38 and 53 seconds, respectively, compared with NRM. This is a significant finding, as a slower decline in ORI suggests a longer time before the onset of hypoxemia. A previous study in pediatric patients^39^ has shown that ORI monitoring can detect a reduction in oxygenation approximately 30 seconds before a rapid drop in Spo_2_.

### Limitations

This study had several limitations. First, the inclusion of only lung-healthy participants restricts the applicability of the findings to patients with underlying lung pathology, who may respond differently to preoxygenation strategies. Second, the controlled study environment does not fully replicate the complexities encountered in clinical conditions, such as patient agitation, difficulties in achieving a tight mask seal, or the variability of prehospital and emergency settings. Third, and most important, the study did not measure the duration of safe apnea following preoxygenation, which is the most clinically relevant end point for evaluating preoxygenation efficacy. Additionally, effective PEEP was not directly measured, meaning that in cases of air leak, the actual PEEP applied may have differed. However, we ensured a proper mask seal by confirming the characteristic hissing sound from the valve. Addressing these limitations in future studies could yield more comprehensive and clinically applicable insights.

## Conclusions

This crossover randomized clinical trial underscores the inferiority of NRM for preoxygenation and demonstrates significantly lower Feo_2_ levels compared with both BVM and BVM plus PEEP. Additionally, BVM plus PEEP outperformed BVM without PEEP by achieving higher Feo_2_ levels in adults and improving ventilation in the posterior lung regions during preoxygenation. These findings support the integration of BVM plus PEEP for preoxygenation into standard care protocols in emergency medicine.

## Notes

Supplement 1.
Trial Protocol

Supplement 2. **eTable 1.** Effect of the 3 Preoxygenation Devices on Feo_2_ at the End of the Preoxygenation Phase **eTable 2.** Effect of the 3 Preoxygenation Devices on Ventilation in Dependent Lung Regions at the End of Preoxygenation **eTable 3.** Effect of the 3 Preoxygenation Devices on Oxygen Reserve Index at the End of the Preoxygenation Phase **eTable 4.** Effect of the 3 Preoxygenation Devices on Time Needed for Oxygen Reserve Index to Return to Baseline After the Preoxygenation Phase **eFigure.** CONSORT Flow Diagram

Supplement 3.
Data Sharing Statement
