# Supplementary material for: Preoxygenation With and Without Positive End-Expiratory Pressure in Lung-Healthy Volunteers: A Randomized Clinical Trial
Source: JAMA Netw Open. 2025 May 20;8(5):e2511569. doi: 10.1001/jamanetworkopen.2025.11569 (PMC12093187; doi:10.1001/jamanetworkopen.2025.11569)
Supplement: Supplement 1. — Trial Protocol [file jamanetwopen-e2511569-s001.pdf]

Study protocol

Title:

PREOX

Optimal preoxygenation strategy during pre-hospital emergency anesthesia"

Type of study:

Interventional, non-pharmacological, randomized, cross-over study in volunteers.

Notice for the obligation of secrecy

The information contained in this protocol is strictly confidential and is submitted to the ethics committee for evaluation and control. Publication of this document without written consent is prohibited and is only authorized if a participant in the study has given permission. After approval of this protocol, its rules will be mandatory for all participants.

Protocol for the PRE-OX study

Study title:

"PRE-OX: optimal preoxygenation strategy during pre-hospital emergency anesthesia"

Running title:

"PRE-OX"

Index

1 Formalia of the PREOX study 5

1.1 Title of the project 5

1.2 Project coordinators 5

1.3 Organising Institution 5

1.4 Partner Institutions 5

1.5 Involved researchers (in alphabetic order) 5

1.6 Medical responsibility and assistance 5

1.7 Date and location of the study 6

2 Summary of the PRE-OX study 6

3 Description of the PRE-OX Study 7

3.1 Introduction 7

3.2 Study hypothesis 8

3.3 Study objective 8

3.4 Possible benefits of the study 9

4 PRE-OX Study protocol 9

4.1 Study type 9

4.2 Sample size calculation 9

The sample size was calculated based on an estimated effect size of 10 and a standard deviation of 8, derived from a previous study.22 Using a power of 0.8 and a significance level of 0.05, and accounting for the repeated measures design with an intra-class correlation (ρ) of 0.3, the required sample size was determined to be 15 participants per group. 9

4.3 Inclusion criteria 10

4.4 Exclusion criteria 10

4.5 Devices used during the study 10

Devices used to perform pre-oxygenation: 10

Devices used to to assess the primary and secondary outcomes: 10

5 Course of the PRE-OX study 11

5.1. Study method 11

5.2 Interruption of the study by the participants 12

6 Ethical and legal foundations 13

6.1 Information and informed consent 14

6.2 Criteria for withdrawing from the study 14

6.3 Risks for participants 14

6.4 Benefits for Participants 14

6.5 Participant insurance 15

7 Conflict of interest 15

8 Financing 15

9 Literature 16

1 Formalia of the PREOX study

1.1 Title of the project

"PRE-OX: optimal preoxygenation strategy during pre-hospital emergency anesthesia"

1.2 Project coordinators

Simon Rauch, MD, PhD[1,2]
Giulia Roveri, MD[1,2]

1.3 Organising Institution

Institute of mountain emergency medicine, Eurac Research
Via Ipazia 2
I-39100 Bolzano
Italy

1.4 Partner Institutions

Medical Faculty of University Ulm
Albert-Einstein-Allee 11
89081 Ulm

Aiut Alpin Dolomites Mountain Helicopter Rescue Service
I-39040 Pontives/Laion (BZ)

1.5 Involved researchers (in alphabetic order)

Hermann Brugger, MD[1]; Anna Camporesi; Marie Dieß[3]; Alex Hofer, MD[4]; Björn Hossfeld, MD[5]; Simon Kahlen[6]; Giacomo Strapazzon, MD, PhD[1, A]

1.6 Medical responsibility and assistance

Simon Rauch, MD, PhD[1,2]; Giulia Roveri, MD[1,2]

[1] Institute of Mountain Emergency Medicine, Eurac research, Via Ipazia 2, 39100 Bolzano, Italy
[2]Department of Anesthesiology and Intensive Care Medicine, "F. Tappeiner" Hospital, 39012 Merano, Italy
[3]Paracelsus Medical University Salzburg, Strubergasse 21, 5020 Salzburg, Österreich
[4]Aiut Alpin Dolomites, Pontives, 39040, Italy
[5]Klinik für Anästhesiologie, Intensivmedizin, Notfallmedizin und Schmerztherapie Bundeswehrkrankenhaus Ulm Oberer Eselsberg 40 89081 Ulm, Germany

[6]Medical Faculty of University Ulm, Albert-Einstein-Allee 11, 89081 Ulm, Germany

1.7 Date and location of the study

The study will take place in spring-autumn 2024 at the terraXcube of Eurac Research in the Province of Bolzano, Italy.

The PRE-OX Study

2 Summary of the PRE-OX study

Title	PRE-OX: optimal preoxygenation strategy during pre-hospital emergency anesthesia
Study participants	Healthy weight adults, overweight and obese adults (BMI >25 kg/m^2) and children aged 6-12 years
Study design	Interventional, non-pharmacological, randomized, controlled, cross-over study in volunteers
Study objective	Comparing the efficacy of three different preoxygenation strategies, i.e. non-rebreather face mask, BVM with and BVM without additional PEEP (10 mbar) in three subgroups of spontaneously breathing volunteers
Study endpoints	**Primary Endpoint:**<br>The difference in FeO_2 after 3 min of preoxygenation<br>**Secondary endpoints:**<br>- Changes in regional ventilation within the posterior lung regions from baseline to the end of preoxygenation<br>- Differences in ORI at the end of preoxygenation<br>- Time to baseline ORi after the preoxygenation
Number of participants	15 participants per subgroup, i.e. 45 participants in total.
Study date	Spring-Autumn 2024

Study location	terraXcube, Eurac Research - Bolzano, Italy
Inclusion criteria	- Normal-weight adults (BMI 18.5-24.9 kg/m^2) with an „American Society of Anesthesiologists Physical Status Classification System (ASA)“ score of I or II<br>- Adults with a BMI 25-39.9 kg/m^2 with and ASA score <3<br>- Healthy (ASA I) children aged 6-12 years
Exclusion criteria	ASA 3, Age < 6 and age 12-18, pregnant women, missing informed consent, signs and symptoms of an acute respiratory illness on the study day.
Short description of the study	After informed consent and a medical check-up, baseline measurements will be done for 10 minutes (SpO_2, ORI, regional ventilation). Then the participants will undergo 3 different preoxygenation sessions with the 3 interfaces (i.e. non-rebreather facemask with reservoir and a bag-valve-mask with and without PEEP) in a randomized order and a 30 min washout between the sessions. Each preoxygenation session is conducted in a supine position and will be proceeded for 3 min. Afterwards 10 min of SpO_2, ORI and regional ventilation measurements will follow.

3 Description of the PRE-OX Study

3.1 Introduction

Maintaining optimal oxygenation during pre-hospital emergency anesthesia (PHEA) is crucial. Pre-oxygenation involves administering high concentrations of oxygen to patients before anesthesia induction. The goal is to replace the nitrogen normally present in the lungs with oxygen. This increases the patient's oxygen reserves, allowing them to withstand a longer apnea duration. This period refers to the time from apnea onset due to anesthesia until resumption of oxygenation and ventilation following airway management. Optimal pre-oxygenation enhances anesthesia induction safety by reducing hypoxia-related complications such as cardiac arrest, especially crucial in out-of-hospital emergencies where airway management is challenging and time-consuming.(1)

Multiple pre-oxygenation strategies exist for PHEA, yet the optimal technique remains unclear.(2) The PREOX-survey in the UK revealed a wide variation in pre-oxygenation

strategies and that pre-oxygenation is most frequently delivered by bag-valve-mask (BVM) without positive end-expiratory pressure (PEEP) or non-rebreather face masks.(3)

3.2 Study hypothesis

We hypothesize that incorporating a positive end-expiratory pressure (PEEP) of 10 mbar during preoxygenation could enhance efficacy by augmenting the lung's functional residual capacity (FRC), i.e. the volume of air that remains in the lungs after a normal expiration. During anesthesia induction, especially in children and obese individuals, the FRC plays a crucial role in maintaining adequate oxygenation and preventing complications related to respiratory function. In children, FRC is relatively lower compared to adults due to their smaller lung size and higher metabolic rate.(4, 5) During anesthesia induction, there's a risk of rapid desaturation if FRC is not optimized. Obesity can lead to reduced FRC due to increased abdominal pressure, decreased lung compliance, and altered respiratory mechanics.(6) This predisposes obese patients to rapid desaturation during anesthesia induction.

3.3 Study objective

We aim at comparing the efficacy of three different preoxygenation strategies, i.e. non-rebreather face mask, BVM with and BVM without additional PEEP in three spontaneously breathing subgroups of volunteers, i.e. healthy normal weight adults, overweight adults (BMI >25 kg/m^2) and children aged 6-12 years. We hypothesize that the different strategies have different pre-oxygenation efficacy both within and between these participant subgroups.

Primary Endpoint:
The primary endpoint is the difference in FeO_2 after 3 min of preoxygenation.

Secondary endpoints:

- Changes in regional ventilation (measured with EIT) within the posterior lung regions from baseline to the end of preoxygenation
- Differences in Oxygen Reserve Index (ORI) at the end of preoxygenation
- Time to basline ORi after the preoxygenation

The ORi is a non-invasive and continuous parameter intended to provide insight into a patient's oxygen status in the moderate hyperoxic range (PaO_2 >100 and ≤200 mmHg). https://professional.masimo.de/technology/co-oximetry/ori/.

Electrical impedance tomography (EIT) quantifies ventilation in different lung regions by measuring changes in electrical impedance across the thorax. During ventilation, air content in the lungs alters impedance to electrical currents differently depending on the ventilation distribution within the lungs. EIT uses an array of electrodes placed around the chest to pass small alternating electrical currents through the thorax. These currents generate impedance measurements that are used to reconstruct images showing changes in regional ventilation distribution over time.

3.4 Possible benefits of the study

Considering the conflicting findings regarding the optimal preoxygenation method in the prehospital setting, and the lack of high-quality studies involving children and obese subjects, this study has the potential to shed light on the issue and establish the most effective pre-oxygenation strategy in prehospital care.

4 PRE-OX Study protocol

4.1 Study type

Interventional, non-pharmacological, randomized, controlled, cross-over study in volunteers.

4.2 Sample size calculation

The sample size was calculated based on an estimated effect size of 10 and a standard deviation of 8, derived from a previous study.22 Using a power of 0.8 and a significance level of 0.05,

and accounting for the repeated measures design with an intra-class correlation (ρ) of 0.3, the required sample size was determined to be 15 participants per group.

4.3 Inclusion criteria

- Normal-weight adults (BMI 18.5-24.9 kg/m2) with an „American Society of Anesthesiologists Physical Status Classification System (ASA) " Score of I or II
- Adults with a BMI 25-39.9 kg/m2with an ASA score <3
- Healthy (ASA I) children aged 6-12 years

4.4 Exclusion criteria

- ASA ≥3
- Age < 6 and age 13-18
- Pregnant women
- Missing informed consent
- Signs and symptoms of an acute respiratory illness on the study day

4.5 Devices used during the study

Devices used to perform pre-oxygenation:

- Non-rebreather facemask with reservoir
- BVM without PEEP valve
- BVM with PEEP valve

Devices used to to assess the primary and secondary outcomes:

- 'RD rainbow Lite SET-1' fingertip sensor and 'Radical-7® Pulse CO-Oximeter®' monitor (Masimo Corporation, Irvine, CA, USA) to measure the ORi
- Dräger X-am 5600 for FeO_2 measurement
- Electrical impedance tomograph (EIT) (ENLIGHT, Timpel Medical, Eindhoven, NL)

5 Course of the PRE-OX study

5.1. Study method

On arrival of the participants, a medical interview with a general medical examination will be performed, including measurement of blood pressure, heart rate, heart and lung auscultation. Thereafter, the informed consent will be obtained. Next, the participant will be equipped with the monitoring devices, i.e., finger sensor for SpO_2 and ORi and EIT chest belt. Baseline measurements for SpO_2, ORi and regional lung ventilation will be performed in a supine position. The spontaneously breathing participants will than undergo three pre-oxygenation sessions, i.e., with a non-rebreather face mask, a BVM with a PEEP of 8 mbar and without PEEP, each additionally equipped with an O_2 reservoir and with an O_2 flow of 15 l/min, in a crossover design and with randomized order. The preoxygenation sessions will be performed in a supine position and terminated after three minutes. After each pre-oxygenation session the participants will remain in the supine position and a follow up measurement of SpO_2, ORi and regional ventilation will be done for 10 minutes. A 30-minute wash out period will follow before the next preoxygenation session. The study ends after running through all three preoxygenation sessions.

If participants provide informed consent, the study will involve recording audio and video

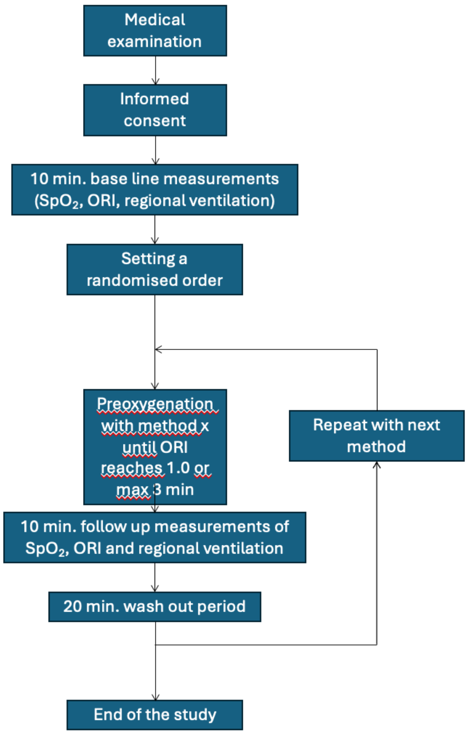

material.

Figure 1:Study protocol. SpO_2 (Peripheral Oxygen Saturation), ORI (Oxygen Reserve Index), FeO_2: expiratory O_2-concentration.

5.2 Interruption of the study by the participants

The participants can interrupt the study for any reason and at any time.

6 Ethical and legal foundations

The study complies with the Helsinki Declaration and subsequent amendments, the Convention on

Human Rights and Biomedicine and the Italian Law on Research.

Participants will be informed about the aims and perspectives of the study. To take part in the research, they will have to sign a complete informed consent form containing detailed information. The participant, if capable of understanding and willing, will be free to withdraw his/her consent to participate at any time by telephone and confirming it in writing.

The data processing will be compliant with the EU Regulation 2016/679 (GDPR) and with the Legislative Decree n. 196/2003, concerning the protection of individuals regarding the processing of personal data.

The right holder of the data processing is Eurac Research. Responsible for the scientific part is the project coordinator (Prof. Simon Rauch, MD PhD).

All acquired data are electronically stored in coded forms only. From these codes it will be impossible to gain information regarding the identity of the participants. The key to the codes will only be accessible to the researchers directly involved in the study. Similarly, study results will be published in scientific journals or at conferences in a form that makes it impossible to uncover the identity of the participants.

Specifically, the personal data of the participants will be processed exclusively by researchers authorized by the data controller and the processing will be based on the respect of the principles of correctness, lawfulness, and transparency as well as protection of confidentiality. Personal data will be used exclusively for scientific research purposes.

6.1 Information and informed consent

Study participants will be informed written and orally of the risks, objectives, and possible developments, as well as the protection and processing of personal data that will be collected as part of participation in the research study, specifying the voluntary nature of participation and the possibility to withdraw from the study at any time.

6.2 Criteria for withdrawing from the study

Participants can withdraw from the study at any time and, in this case, the collected data of the subject in question will be destroyed pursuant to art. 17 GDPR, except for what was established in the art. 17, paragraph 3 GDPR. Further information regarding the early termination of the study is reported in paragraph 5.4.

6.3 Risks for participants

Everything planned for this trial is designed with the primary aim of obtaining maximum safety for the participant. All the planned measurements are routinely used in research and clinical practice. Firmly pressing the face mask over the participant's nose and mouth to ensure a tight fit could cause slight discomfort or panicking. All used measurements are non-invasive procedures. In all tests these risks will be further mitigated by having an emergency doctor and emergency medical kit on site.

6.4 Benefits for Participants

For study participants, no profit is foreseen, meaning no remuneration is provided apart from reimbursement for travel and meal. At the end of the study, participants will receive feedback on the study. If requested, participants can be informed of their individual test results once the data has been extracted and analysed at the end of the study. The participants will benefit of a free medical examination.

6.5 Participant insurance

The physicians, researchers and participants in the study will be covered by an insurance policy, specifically written for this study. This policy will cover liability to third parties for any unintentional damage to health and/or financial loss arising therefrom, as well as violation of privacy rights. This policy will cover any damages suffered directly or indirectly by the participant as a result of interventions undertaken in connection with the study.

7 Conflict of interest

The objective of the project is clinical research without any monetary profit from the study participants or their family members. The emergency medical services involved have no influence on the design and development of the study, the detection and processing of scientific data, or the drafting of scientific publications.

The investigators certify that they have no conflict of interest or involvement in any organization or entity with any financial or non-financial interest in the materials analysed in this study.

8 Financing

The study will be supported by the Institute of Mountain Emergency Medicine of Eurac Research.

9 Literature

1. Pietsch U, Knapp J, Kreuzer O, Ney L, Strapazzon G, Lischke V, et al. Advanced airway management in hoist and longline operations in mountain HEMS - considerations in austere environments: A narrative review This review is endorsed by the International Commission for Mountain Emergency Medicine (ICAR MEDCOM). Scandinavian Journal of Trauma, Resuscitation and Emergency Medicine. 2018;26(1).

2. Bailly A, Ricard JD, Le Thuaut A, Helms J, Kamel T, Mercier E, et al. Compared Efficacy of Four Preoxygenation Methods for Intubation in the ICU: Retrospective Analysis of McGrath Mac Videolaryngoscope Versus Macintosh Laryngoscope (MACMAN) Trial Data. Crit Care Med. 2019;47(4):e340-e8.

3. Boulton AJ, Mashru A, Lyon R. Oxygenation strategies prior to and during prehospital emergency anaesthesia in UK HEMS practice (PREOXY survey). Scandinavian journal of trauma, resuscitation and emergency medicine. 2020;28(1):99.

4. Di Cicco M, Kantar A, Masini B, Nuzzi G, Ragazzo V, Peroni D. Structural and functional development in airways throughout childhood: Children are not small adults. Pediatr Pulmonol. 2021;56(1):240-51.

5. Gander S, Frascarolo P, Suter M, Spahn DR, Magnusson L. Positive end-expiratory pressure during induction of general anesthesia increases duration of nonhypoxic apnea in morbidly obese patients. Anesth Analg. 2005;100(2):580-4.

6. Salome CM, King GG, Berend N. Physiology of obesity and effects on lung function. Journal of applied physiology (Bethesda, Md : 1985). 2010;108(1):206-11.
