# Supplementary material for: Preoxygenation With and Without Positive End-Expiratory Pressure in Lung-Healthy Volunteers: A Randomized Clinical Trial
Source: JAMA Netw Open. 2025 May 20;8(5):e2511569. doi: 10.1001/jamanetworkopen.2025.11569 (PMC12093187; doi:10.1001/jamanetworkopen.2025.11569)
Supplement: Supplement 2. — eTable 1. Effect of the 3 Preoxygenation Devices on Feo2 at the End of the Preoxygenation Phase eTable 2. Effect of the 3 Preoxygenation Devices on Ventilation in Dependent Lung Regions at the End of Preoxygenation eTable 3. Effect of the 3 Preoxygenation Devices on Oxygen Reserve Index at the End of the Preoxygenation Phase eTable 4. Effect of the 3 Preoxygenation Devices on Time Needed for Oxygen Reserve Index to Return to Baseline After the Preoxygenation Phase eFigure. CONSORT Flow Diagram [file jamanetwopen-e2511569-s002.pdf]

Supplementary Online Content

Roveri G, Camporesi A, Hofer A, Kahlen S, Breidt F, Rauch S. Preoxygenation with and without positive end-expiratory pressure in lung-healthy volunteers: a randomized clinical trial. *JAMA Netw Open.* 2025;8(5):e2511569. doi:10.1001/jamanetworkopen.2025.11569

eTable 1. Effect of the 3 Preoxygenation Devices on Feo_2 at the End of the Preoxygenation Phase

eTable 2. Effect of the 3 Preoxygenation Devices on Ventilation in Dependent Lung Regions at the End of Preoxygenation

eTable 3. Effect of the 3 Preoxygenation Devices on Oxygen Reserve Index at the End of the Preoxygenation Phase

eTable 4. Effect of the 3 Preoxygenation Devices on Time Needed for Oxygen Reserve Index to Return to Baseline After the Preoxygenation Phase

eFigure. CONSORT Flow Diagram

This supplementary material has been provided by the authors to give readers additional information about their work.

© 2025 Roveri G et al. *JAMA Network Open*.

eTable 1. Effect of the 3 Preoxygenation Devices on FeO_2 at the End of the Preoxygenation Phase

Results are expressed as pairwise comparisons between the devices obtained after fitting a linear mixed model on FeO_2 (details in methods section) and reported with their relative 95% CI and p values.
FeO_2: Expired Fraction of Oxygen; NRM: nonrebreather mask; BVM: bag-valve mask; BVM plus PEEP: bag-valve mask with positive end-expiratory pressure.

		Contrast	std. err.	z	P>z	CI
Normal weight adults (NW)	BVM vs NRM	19.6	2.03	9.65	<0.001	15.6 to 23.6
	BVM plus PEEP vs NRM	23.1	1.71	13.49	<0.001	19.8 to 26.5
	BVM plus PEEP vs BVM	3.5	1.65	2.13	0.03	0.3 to 6.8
Overweight/obese (OW-OB)	BVM vs NRM	13.8	2.31	5.98	<0.001	9.3 to 18.4
	BVM plus PEEP vs NRM	21.0	1.80	11.64	<0.001	17.5 to 24.6
	BVM plus PEEP vs BVM	7.2	2.58	2.78	0.005	2.1 to 12.3
Children	BVM vs NRM	26.5	2.97	8.89	<0.001	21.1 to 32.4
	BVM plus PEEP vs NRM	29.4	2.65	11.07	<0.001	23.9 to 34.5
	BVM plus PEEP vs BVM	2.9	2.88	1.00	0.32	-3.0 to 8.0

eTable 2. Effect of the 3 Preoxygenation Devices on Ventilation in Dependent Lung Regions at the End of Preoxygenation

Results are expressed as pairwise comparisons between the devices obtained after fitting a linear mixed model on dependent lung ventilation (details in methods section) and reported with their relative 95% CI and p values.
EIT: Electrical Impedance Tomography; NRM: nonrebreather mask; BVM: bag-valve mask; BVM plus PEEP: bag-valve mask with positive end-expiratory pressure.

		Contrast	std. err.	z	P>z	CI
Normal weight adults (NW)	BVM vs NRM	2.0	1.1	1.30	0.015	0.3 to 3.6
	BVM plus PEEP vs NRM	4.9	2.2	4.95	0.026	0.6 to 9.2
	BVM plus PEEP vs BVM	2.9	1.8	3.06	0.002	2.0 to 9.1
Overweight/obese (OW-OB)	BVM vs NRM	-1.0	1.8	-0.58	0.588	-4.6 to 2.6
	BVM plus PEEP vs NRM	5.3	2.6	1.05	<0.001	2.2 to 8.4
	BVM plus PEEP vs BVM	3.9	1.0	2.48	<0.001	1.8 to 5.9
Children	BVM vs NRM	3.2	1.4	2.62	0.03	0.3 to 6.1
	BVM plus PEEP	5.3	1.7	3.00	0.002	1.9 to 8.7

© 2025 Roveri G et al. *JAMA Network Open*.

	vs NRM					
	BVM plus PEEP vs BVM	2.0	1.7	1.34	0.271	-1.2 to 5.4

eTable 3. Effect of the 3 Preoxygenation Devices on Oxygen Reserve Index at the End of the Preoxygenation Phase

Results are expressed as pairwise comparisons between the devices obtained after fitting a linear mixed model on ORI (details in methods section) and reported with their relative 95% CI and p values.
ORI: Oxygen Reserve Index; NRM: nonrebreather mask; BVM: bag-valve mask; BVM plus PEEP: bag-valve mask with positive end-expiratory pressure.

		Contrast	std. err.	z	P>z	CI
Normal weight adults (NW)	BVM vs NRM	-0.01	0.03	-0.36	0.72	-0.0 to -0.05
	BVM plus PEEP vs NRM	0.03	0.05	0.74	0.46	-0.05 to 0.12
	BVM plus PEEP vs BVM	0.05	0.04	1.27	0.21	-0.03 to 0.11
Overweight/obese (OW-OB)	BVM vs NRM	-0.02	0.03	-0.60	0.55	-0.07 to 0.04
	BVM plus PEEP vs NRM	0.06	0.02	3.66	<0.001	0.02 to 0.09
	BVM plus PEEP vs BVM	0.04	0.01	2.03	0.04	0.00 to 0.07
Children	BVM vs NRM	0.01	0.02	0.77	0.44	-0.02 to 0.04
	BVM plus PEEP vs NRM	0.03	0.02	1.33	0.18	-0.01 to 0.06
	BVM plus PEEP vs BVM	0.01	0.01	0.87	0.39	-0.01 to 0.04

eTable 4. Effect of the 3 Preoxygenation Devices on Time Needed for Oxygen Reserve Index to Return to Baseline After the Preoxygenation Phase

Results are expressed as pairwise comparisons between the devices used obtained after fitting a linear mixed model on time (details in methods section) and reported with their relative 95% CI and p values.
ORI: Oxygen Reserve Index; NRM: nonrebreather mask; BVM: bag-valve mask; BVM plus PEEP: bag-valve mask with positive end-expiratory pressure.

		Contrast	std. err.	z	P>z	CI
Normal weight adults (NW)	BVM vs NRM	2.6	2.4	0.11	0.91	-44.9 to 50.2
	BVM plus PEEP vs NRM	34.1	2.6	1.27	0.20	-18.4 to 86.7
	BVM plus PEEP vs BVM	31.5	2.4	1.28	0.20	-16.7 to 79.7
Overweight/ Obese (OW-OB)	BVM vs NRM	27.4	1.7	1.59	0.11	-6.3 to 61.0

© 2025 Roveri G et al. *JAMA Network Open*.

	BVM plus PEEP vs NRM	38.2	1.4	2.59	0.01	9.3 to 67.2
	BVM plus PEEP vs BVM	10.9	1.3	0.80	0.42	-15.1 to 37.6
Children	BVM vs NRM	19.0	1.1	1.70	0.09	-3.0 to 41.0
	BVM plus PEEP vs NRM	53.2	16.6	10.2	<0.001	19.8 to 86.5
	BVM plus PEEP vs BVM	31.8	16.4	5.19	0.008	1.0 to 64.8

© 2025 Roveri G et al. *JAMA Network Open*.

eFigure. CONSORT Flow Diagram

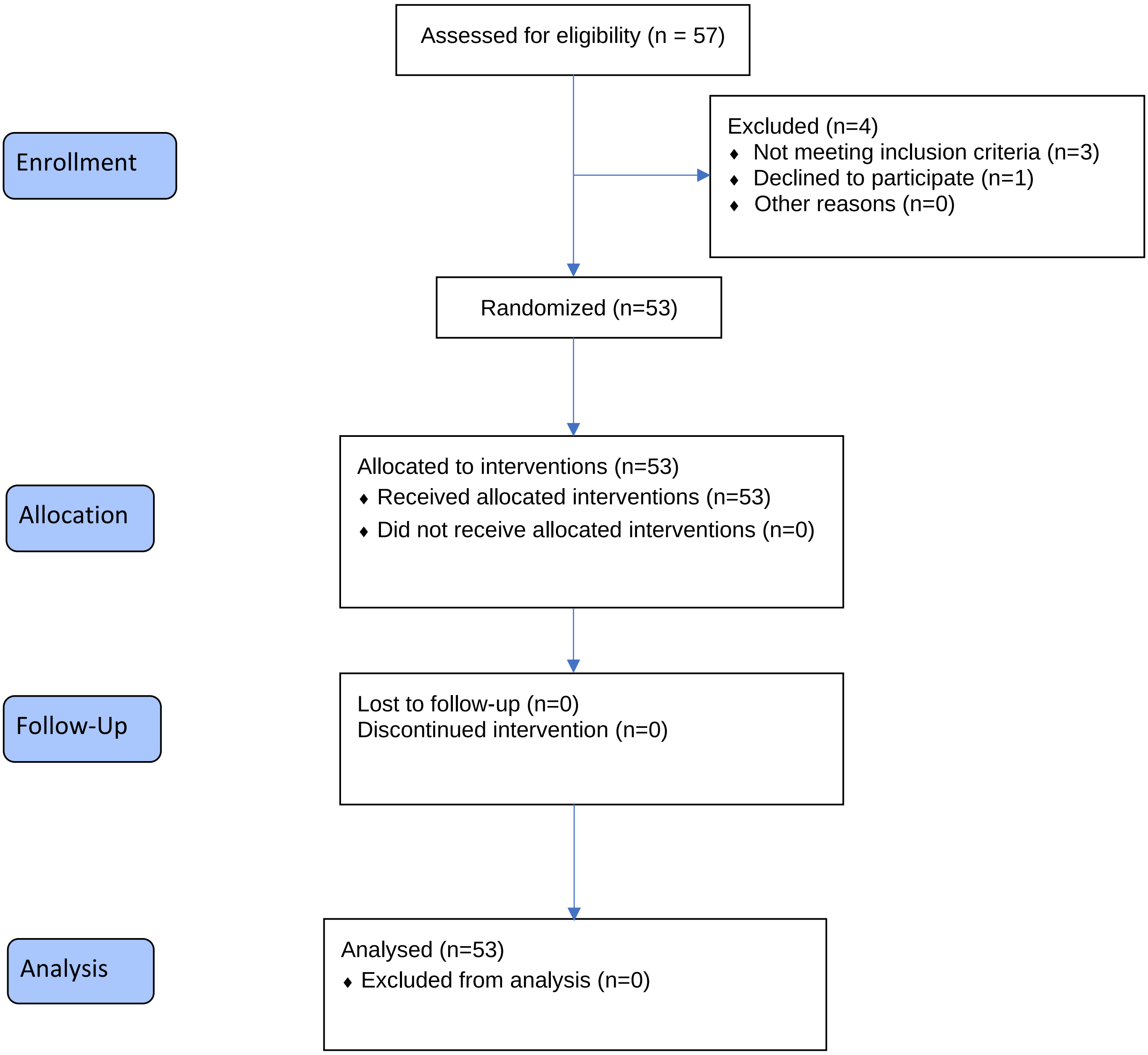

© 2025 Roveri G et al. *JAMA Network Open*.
