# Supplementary material for: Preoxygenation With and Without Positive End-Expiratory Pressure in Lung-Healthy Volunteers: A Randomized Clinical Trial
Source: JAMA Netw Open. 2025 May 20;8(5):e2511569. doi: 10.1001/jamanetworkopen.2025.11569 (PMC12093187; doi:10.1001/jamanetworkopen.2025.11569)
Supplement: Supplement 3. — Data Sharing Statement [file jamanetwopen-e2511569-s003.pdf]

Data Sharing Statement

Roveri. Preoxygenation With and Without Positive End-Expiratory Pressure in Lung-Healthy Volunteers. *JAMA Netw Open*. Published May 20, 2025.
doi:10.1001/jamanetworkopen.2025.11569

Data
Additional Information: Registration at ClinicalTrials.gov (NCT06370689) before recruitment of the first participant.
Data available: No

Additional Information
Explanation for why data not available: Data will be provided by the authors upon reasonable request.
